# Internal limiting membrane peeling combined with mouse nerve growth factor injection for idiopathic macular hole

**DOI:** 10.1186/s12886-023-03066-1

**Published:** 2023-07-31

**Authors:** Xiao Yu, Lingyao Wu, Ziqing Mao, Huimin Fan, Wenjia Dong, Zhipeng You

**Affiliations:** The Affiliated Eye Hospital of Nanchang University, Jiangxi Province Ocular Disease Clinical Research Center, Jiangxi Institute of Ophthalmology and Vision Science, Jiangxi Provincial Key Laboratory of Ophthalmology, 330006 Nanchang, China

**Keywords:** Idiopathic macular hole, Internal limiting membrane peeling, Mouse nerve growth factor, Mean retinal sensitivity recovery

## Abstract

**Background:**

The study was intended to confirm whether Pars Plana Vitrectomy (PPV) with Internal Limiting Membrane (ILM) peeling and intravitreal injection mouse Nerve Growth Factor(mNGF) was effective for the treatment of Idiopathic Macular Hole(IMH) by Optical Coherence Tomography Angiography(OCTA) and microperimetry.

**Methods:**

A retrospective study was performed in adults’ patients. A total of 44 eyes (March 2021-October 2021) with IMH who received surgical treatment in the Affiliated Eye Hospital of Nanchang University in Nanchang City, Jiangxi Province were selected. The subjects were treated using PPV combined with ILM peeling and intravitreal mNGF (combined group) or PPV combined with ILM peeling (placebo group). The Best Corrected Visual Acuity (BCVA), Optical Coherence Tomography Angiography (OCTA) and MP-3 microperimetry were carried out and observed at baseline, 1 week(1W), 1,3 and 6 months (1 M,3 M,6 M) postoperatively.

**Results:**

The minimum diameter of MH were (568.650 ± 215.862)μm and (533.348 ± 228.836)μm in the Placebo and Combine group pre-operative. During the observation, the macular hole closure rate in the placebo group and combined group were 90% and 95.8% respectively and the difference was not statistically significant(*p* = 0.583). Compared to pre-surgery, the perimeter and circularity of Foveal Avascular Zone (FAZ) in the placebo group decreased at 1,3,6 M (*p* = 0.001, < 0.001, < 0.001) and 1W,1,6 M (*p* = 0.045,0.010, < 0.001) post-surgery respectively. And the perimeter and circularity of FAZ showed significant reduction in the combined group at 1,3,6 M (*p* = 0.005,0.004, < 0.001) and at each follow-up time point (all values of* p* < 0.001). The vascular density of SCP increased at 1W(*p* = 0.031) and 6 M(*p* = 0.007), the perfusion density of SCP was significantly improved at each follow-up time point (*p* = 0.028, 0.011, 0.046, 0.004) in the combined group. The BCVA in the combined group was more obvious than that in the placebo group at 1 M, 3 M and 6 M after operation (t_1_ = 2.248, *p*_*1*_ = 0.030; t_3_ = 3.546, *p*_*3*_ = 0.001; t_6_ = 3.054, *p*_*6*_ = 0.004). The changes of BCVA in the combined group was more conspicuous than that in the placebo group at each follow-up time point, and the difference was statistically significant (t_1_ = 2.206,*p*_*1*_ = 0.033;t_2_ = 2.54,*p*_*2*_ = 0.015;t_3_ = 3.546,*p*_*3*_ = 0.001;t_6_ = 3.124,*p*_*6*_ = 0.003).At 1 M, 3 M and 6 M, the MRS of 2° and 4° in the combined group was better than that in the placebo group(t = -2.429,-2.650,-3.510,-2.134,-2.820,-3.099 *p* = 0.020,0.011,0.001,0.039,0.007,0.004). During various time points, the MRS of 12°in the combined group was better than that in the placebo group, the difference was statistically significant (t = -3.151, -3.912, -4.521, -4.948, *p*_*1*_ = 0.003, < 0.001, < 0.001 < 0.001). The integrity of External Limiting Membrane (ELM) in combination group was better than that in placebo group at 6 M postoperative(*p* = 0.022) and that of Ellipsoid Zone(EZ) was preferable in the combined group at 3 M and 6 M after surgery(*p* = 0.012,0.004). Correlation analysis showed that the integrity of EZ was correlated with 12°MRS at 1 M, 3 M and 6 M after surgery(*r* = -0.318, -0.343,-0.322;*p* = 0.023,0.033, < 0.001). There was no correlation between postoperative ELM integrity and postoperative BCVA and 12°MRS(*p* > 0.05).

**Conclusions:**

Our results manifested that PPV combined with ILM peeling and intravitreal injection mNGF might be more effective for initial IMH. This method increased the blood flow, MRS and promoted the recovery of ELM and EZ in the macular and might improve the visual function of patients postoperatively.

## Background

Idiopathic Macular Hole (IMH), which occurs in the absence of other retinal diseases, is a retinal nerve epithelial layer defect of unknown etiology. Although the exact mechanism of action is unclear, it is widely believed that the traction of the vitreous cortex and Internal Limiting Membrane (ILM) plays an important role in the formation of IMH. It is suggested that the tangential traction leads to the dysfunction of Müller cells and the rupture of Müller cell cones in the fovea [1, 2]. At present, Pars Plana Vitrectomy (PPV) combined with ILM peeling is the main treatment for IMH. Previous studies have shown that the closure rate of macular hole (MH) is 85%-100% after PPV for IMH and the closure rate of IMH is significantly improved post PPV combined with ILM peeling. However, postoperative recovery of visual function is not associated with the improvement of hole closure rate [3–5]. Herein, improving postoperative visual function remains a key topic of research in the field of fundus surgery. Previous studies have reported that mouse Nerve Growth Factor(mNGF) can regulate cell cycle progression, promote Müller cell proliferation, and photoreceptor cell regeneration [6, 7]. Recently, some scholars have proposed that mNGF may promote the postoperative recovery of visual function of IMH. Research has shown that combining PPV with ILM peeling and intravitreal injection of mNGF is a safe and effective treatment for large macular holes, improving not only visual acuity but also promoting functional recovery of the External Limiting Membrane (ELM) and Ellipsoid Zone (EZ) [8]. With the ophthalmic imaging technology developed rapidly, Optical Coherence Tomography Angiography (OCTA) has been widely used in ophthalmology due to the advantages of non-invasive, repeatable, safe, and convenient. Kumagai et al. [9, 10] showed that Foveal Avascular Zone (FAZ) decreased after IMH. What’s more, Cheng et al. [11] indicated that the perfusion density of Superficial Capillary Plexus (SCP) and deep capillary plexus in IMH decreased compared to the placebo group. In summary, previous studies have explored the changes of the structure of macular microvascular before and after IMH surgery, but the effects of mNGF on macular microcirculation and the diagnosis results of microperimetry in macular are rarely reported.

The aim of this study was to compare the differences of macular microcirculation and microperimetry in patients with IMH after PPV with ILM peeling with ILM peeling and intravitreal injection mNGF by OCTA and microperimetry, so as to provide a new view and reference for a clinical treatment of IMH.

## Methods

### Experiment design

The study complied with the Declaration of Helsinki and was approved by the Ethics Committee of the Affiliated Eye Hospital of Nanchang University (YLP202012011). Before surgery, all patients were informed of the purpose of surgery and possible complications, and signed informed consent. The study was designed as a retrospective study to analyze the effect of PPV combined with ILM peeling and intravitreal injection mNGF for the treatment of IMH. 44 eyes of 43 patients with IMH (25 females,18 males) who received surgical treatment in the Affiliated Eye Hospital of Nanchang University in Nanchang City, Jiangxi Province from March 2021 to October 2021 were selected. The patients were divided into two groups randomly according to the random number table method: 24 eyes of 23 patients conducted PPV combined with ILM peeling and intravitreal injection mNGF (combined group); 20 eyes of 20 patients underwent PPV combined with ILM peeling (placebo group).

### Inclusion and exclusion criteria

All the participants met the inclusion criteria as follows: (1) All patients had a retinal nerve epithelial layer defect after Spectral Domain Optical Coherence Tomography (SD-OCT) (ZEISS CIRRUS HD^5000^, Germany). (2) The Minimum diameter of MH were greater than 400 μm. (3) The diopter and the axis were less than -6.0DS and 26.5 mm respectively. (4) Patients whose age over 40 years with mild cataracts were included this study. (5) Patients were regularly follow-up with complete data. The exclusion criteria were as follows: (1) Patients had a lamellar macular hole (LMH) or macular pseudoholes by SD-OCT. (2) Patients with a history of eye trauma, eye surgery and intravitreal injection drugs. (3) Patients were diagnosed as secondary MH that were caused by retinal detachment, age-related macular degeneration, central serous chorioretinopathy, idiopathic polypoid chorioretinopathy etc. (4) Patients suffered from glaucoma, uveitis and other fundus diseases. (5) Patients had blurred image.

## Retinal multimodal imaging

Retinal multimodal imaging included 6 × 6 mm OCTA (ZEISS CIRRUS HD^5000^, Germany) with calculation of vascular densities of SCP using Angio-analytics software (CIRRUS™ HD-OCT Review Software) and MP-3 microperimetry (NIDEK, Japan) measuring the Mean Retinal Sensitivity (MRS) in the macular.

### OCTA and SD-OCT examine

SD-OCT scans were obtained over a 6 × 6mm^2^ area centered on the fovea; the scan density was 512 A-scans (horizontal) × 128 B-scans (vertical); The hole diameters were measured by the embedded manual caliper function of the OCT platform. The scan area of OCTA was 6 × 6 mm centered on the macular and the SCP was situated between 3 mm below the ILM to 15 mm below the inner plexiform layer. The foveal (0-1 mm diameter), parafoveal (1-3 mm diameter) and perifovea(3-6 mm) regions of the ETDRS grid were analyzed of the Vessel Density (VD) in the SCP. Retinal microvasculature was analyzed by the automated retinal layer segmentation algorithm that is available on the equipment. The photography quality below 7/10 was eliminated from the research. The integrity of EZ and ELM is expressed as the horizontal length of the associated discontinuous high reflection line in the SD-OCT.

### MP-3 microperimetry examine

All patients underwent microperimetry examination under dim light conditions with dilated pupils. A 40 degrees grid of 45 spots was centered on the macular area. Stimulus size was Goldmann III white spot, with a duration of 200 ms. Background luminance was 31.4asb. The dynamic range of stimulus intensities was between 0 dB that represents the maximum stimulus luminance to 34 dB that corresponds to the minimum stimulus luminance. A 4–2 fast strategy that the span is 2 dB, starting from 0 to 34 dB range, using as a fixation target a single red cross of 1° of spatial width. The examinations were performed using an automatic eye-tracker, and the follow-up pattern was used postoperatively.

OCTA and microperimetry examinations were performed by the same experienced ophthalmic technician.

## Surgical procedure

All procedures were performed by the same experienced fundus surgeon (Professor You). First, general anesthesia was performed for all cases. Second, phacoemulsification and intraocular lens implantation were performed. Third, the core and peripheral vitreous were cleavage using a non-contact wide-angle viewing system (ZEISS Resight 700 Germany) and a conventional 23-gauge three-port procedure. ILM that was stained by 0.025% brilliant blue (Dual, Dorc, Zuiland, the Netherland) for 10 s. ILM that was center on the macular about 2 Disc Diameter (DD) was removed by forceps. In the combined group, mNGF (mouse Nerve Growth Factor for injection, Staidson, Beijing, China, 6ug/ml, 0.06 ml) was injected into MH with a 1 ml syringe. Finally, air or silicon oil were injected to the vitreous according to the diameter of MH. The patients in the placebo group were instructed to maintain the prone position for 2 to 4 weeks after surgery, and the patients in the combined group maintained this supine position for 6 h to promote mNGF to contact with the remaining Müller cells in MH fully, and then converted to the prone position for 2 to 4 weeks.

## Statistical analysis

Demographic and follow-up data were collected. A detailed ophthalmic test including measurement of BCVA, OCTA, MP-3 microperimetry, dilated fundus examination, axial length and Intraocular Pressure (IOP) were performed in all patients. The BCVA was measured with Snellen chart and was converted to logarithm of minimal angle of resolution (Log MAR) for statistical analyses. The MRS was defined as mean retinal sensitivity of 45 points in the macular within 12°. The BCVA, OCTA and MP-3 microperimetry were carried out at baseline, 1 week(1W), 1,3 and 6 months (1 M,3 M,6 M) postoperatively. The SPSS 24.0 software (SPSS, Inc, Chicago, IL) was used for statistical analyses (Table 1).

**Table 1:** Main outcome measures

Primary Outcome Measures	Secondary Outcomes
BCVA	Minimum diameter of MH
2°, 4°and 12°MRS	The Area, Perimeter and Circularity of FAZ
The Vascular and Perfusion Density of SCP	ELM integrity, EZ integrity

*BCVA* Best Corrected Visual Acuity, *MRS* Mean Retinal Sensitivity, *SCP* Superficial Capillary Plexus, *MH* Macular Hole, *FAZ* Foveal Avascular Zone, *ELM* External Limiting Membrane, *EZ* Ellipsoid Zone

Age, minimum diameter of MH, ELM integrity, EZ integrity, BCVA, 2°, 4°, 12°VA, FAZ perimeter and circularity, the vascular and perfusion density of SCP in the foveal were represented by mean (standard deviation). For these data, independent sample t-test was used for these data for among group and repeated measures ANOVA was used to analyze the repeated measurement data, and paired sample t-test was used within group.

Disease course, IOP, FAZ area, the vascular and perfusion density of SCP in total, inner ring and outer ring were represented by median (lower and upper quartiles).For these data, the Mann–Whitney U test was used for among group and Wilcoxon rank-sum test was used for within group comparison.

The closure rate of MH was represented by the rate of adoption (%). Chi-square test was used for the comparison of among group. The values of *p* < 0.05 were considered statistically significant.

## Results

### Demographic and baseline clinical data

The minimum diameter of MH were (568.650 ± 215.862)μm and (533.348 ± 228.836)μm in the Placebo and Combine group pre-operative. There was no significant difference in preoperative baseline characteristics and clinical data between the two groups (*p* > 0.05). Patients’ demographics, preoperative OCTA, and microperimetry data in the two groups are resumed as follow (Table 2). The follow-up time was ≥ 6 months and no ocular and systemic complications occurred during the follow-up. During the observation, the hole that was closed in the placebo group and combined group were 18 eyes (90%) and 23 eyes (95.8%) respectively, and the difference was not statistically significant (*p* = 0.583). One eye had a recurrent MH in the combined group during the follow up. Finally, there were 2 eyes and 1eye used silicon oil as tamponade agent in the placebo and combine group. During the follow-up, no complications such as raised IOP, endophthalmitis and retinal detachment were found. Dobrowolski et al. showed that silicone oil endotamponade in 23-G PPV was associated with an increased risk of IOP at 24-month follow-up [12]. We think this difference may be due to the short observation period and less use of silicone oil for endotamponade in our study.

**Table 2:** Baseline characteristics and clinical data of the patients

	Placebo group($\overline{x }$±s) [M(QL, QU)]	Combine group($\overline{x }$±s) [M(QL, QU)]	t/Z	*p*
Age (y)	64.80 ± 6.910	62.876 ± 7.491	0.879	0.384
Disease course (M)	6(2 ~ 12)	2(1 ~ 2)	0.476	0.634
Minimum diameter of MH (μm)	568.650 ± 215.862	533.348 ± 228.836	0.518	0.607
ELM integrity (μm)	854.550 ± 380.106	755.375 ± 254.596	-1.031	0.308
EZ integriry (μm)	916.250 ± 564.698	734.083 ± 306.866	-1.360	0.181
IOP (mmHg)	13.75(12 ~ 15)	14(13 ~ 15)	0.392	0.695
BCVA (log MAR)	1.025 ± 0.506	1.117 ± 0.483	0.613	0.543
2°MRS (dB)	18.158 ± 8.298	17.942 ± 8.348	0.285	0.933
4°MRS (dB)	18.584 ± 7.834	18.172 ± 7.873	0.172	0.864
12°MRS (dB)	19.890 ± 6.847	22.633 ± 4.588	1.570	0.124
FAZ are(mm^2^)	0.35(0.27 ~ 0.59)	0.59(0.36 ~ 0.63)	1.947	0.052
FAZ perimeter(mm)	2.866 ± 0.746	3.331 ± 0.940	1.760	0.086
FAZ circularity	0.630 ± 1.219	0.633 ± 1.219	0.114	0.910
**The vascular density of SCP**				
Total (mm^−1^)	17.9(15.7 ~ 18.5)	17.35(15.9 ~ 18.1)	1.028	0.304
Foveal (mm^−1^)	8.932 ± 4.712	8.167 ± 3.901	0.582	0.563
Inner ring(mm^−1^)	17.4(15.6 ~ 18.3)	17.0(15.3 ~ 18.2)	0.844	0.398
Outer ring(mm^−1^)	17.8(16.5 ~ 18.9)	17.75(16.15 ~ 18.4)	1.040	0.298
**The perfusion density of SCP**				
Total(mm^−1^)	0.45(0.39 ~ 0.46)	0.42(0.38 ~ 0.45)	1.235	0.217
Foveal(mm^−1^)	0.198 ± 0.115	0.181 ± 0.959	0.591	0.207
Inner ring(mm^−1^)	0.43(0.38 ~ 0.45)	0.39(0.30 ~ 0.44)	1.284	0.199
Outer ring(mm^−1^)	0.46(0.40 ~ 0.48)	0.44(0.40 ~ 0.47)	1.688	0.091

*Placebo group* pars plana vitrectomy and ILM peeling, combined group: pars plana vitrectomy combined with ILM peeling and intravitreal mouse never growth factor. *MH* macular hole, *IOP* intraocular pressure, *BCVA* best-corrected visual acuity, *log MAR* logarithm of minimal angle of resolution, *MRS* mean retinal sensitivity, *FAZ* foveal area zone, *SCP* superficial capillary plexus, *ELM* External Limiting Membrane, *EZ* Ellipsoid Zone. Values are expressed as mean (standard deviation) or median (lower and upper quartiles). The values of *p* < 0.05 was statistically significant

### The changes of macular microvascular

There was no difference for all these relevant parameters such as the vascular and perfusion density of SCP in all regions from ETDRS in macular, the area, perimeter and circularity of FAZ between the placebo and combined group of all patients at each follow-up time point(*p* > 0.05). The perimeter and circularity of FAZ decreased in the two groups with a statistically significant difference (F = 35.164; F = 17.935; F = 10.954; F = 11.198, all values of *p* < 0.001). Compared to pre-surgery, the perimeter and circularity of FAZ in the placebo group decreased at 1,3,6 M (*p* = 0.001, < 0.001, < 0.001) and 1W,1,6 M (*p* = 0.045,0.010, < 0.001) post-surgery respectively. And the perimeter and circularity of FAZ showed significant reduction in the combined group at 1,3,6 M (*p* = 0.005,0.004, < 0.001) and at each follow-up time point (all values of *p* < 0.001). In the placebo group, no statistically significant changes in the vascular and perfusion density of SCP in the foveal region were found (*p* > 0.05), and in the combined group, the vascular density of SCP increased at 1W(*p* = 0.031) and 6 M(*p* = 0.007), the perfusion density of SCP was significantly improved at each follow-up time point (*p* = 0.028, 0.011, 0.046, 0.004).

### The changes of BCVA

**Fig. 1:**
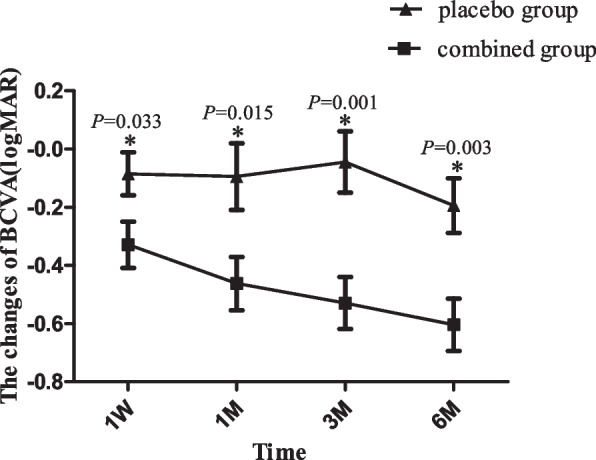
Changes of BCVA after surgery. The changes of BCVA at 1 month,3 month and 6 months after surgical intervention were shown. BCVA: Best Corrected Visual Acuity;log MAR: logarithm of minimal angle of resolution

The difference in time of BCVA between the two groups was statistically significant (F_time_ = 14.128, *p* < 0.001), but the changing trend was different. There were statistically significant in intergroup and interaction in the case of BCVA (F_group_ = 4.165, *p* = 0.048; F_group*time_ = 2.948, *p* = 0.032). The BCVA of the placebo group improved with the time, but the improvement was not statistically significant (all values of *p* > 0.05), and that increased significantly at each follow-up time point in the combined group, the difference was statistically significant (all values of *p* < 0.001). In placebo group, the BCVA was significantly superior at 6 M compared with 3 M(*p* = 0.043). However, the results of each pairwise comparison in terms of BCVA at each follow-up were statistically significant in the combined group(*p* < 0.001,0.004,0.023,0.001). BCVA in the combined group was more obvious than that in the placebo group at 1 M, 3 M and 6 M after operation (t_1_ = 2.248, *p*_*1*_ = 0.030; t_3_ = 3.546, *p*_*3*_ = 0.001; t_6_ = 3.054, *p*_*6*_ = 0.004). The changes of BCVA in the combined group was more conspicuous than that in the placebo group at each follow-up time point, and the difference was statistically significant (t_1_ = 2.206, *p*_*1*_ = 0.033; t_2_ = 2.54, *p*_*2*_ = 0.015; t_3_ = 3.546, *p*_*3*_ = 0.001; t_6_ = 3.124, *p*_*6*_ = 0.003) (Fig. 1).

### The changes of 2° MRS

The difference of 2° MRS in the two groups was statistically significant and there was an interaction effect (F_time_ = 3.757, *p* = 0.011; F_group*time_ = 5.194, *p* = 0.002).The MRS of 2° in the placebo group decreased at each follow-up time point and the difference was statistically significant (*p* = 0.017,0.026,0.004,0.025), and it significantly decreased at 3 M compared with 1 M (*p* = 0.047).However, it was statistically improved at 1 M, 3 M and 6 M (*p* = 0.011,0.016,0.01) in the combined group. Besides, it was inferior in the case of 2° VA at 1W when compared with that at 1 M, 3 M, and 6 M(*p* = 0.002,0.032, < 0.001) and the MRS of 2° at 6 M were superior to that at 1 M and 3 M(*p* = 0.014,0.002), the difference was statistically significant. At 1 M, 3 M and 6 M post-operation, the MRS of 2°in the combined group was significantly superior to that in the placebo group (t_1_ = -2.429, *p*_*1*_ = 0.020; t_3_ = -2.650,* p*_*3*_ = 0.011; t_6_ = -3.510, *p*_*6*_ = 0.001) (Table 3).

**Table 3:** The comparision of MRS between the combined group and placebo group

	2°MRS			4°MRS			12°MRS		
	Combined group	Placebo group	*P*_*1*_	Combined group	Placebo group	*P*_*2*_	Combined group	Palcebo group	*P*_*3*_
1 W	16.084 ± 7.996	19.329 ± 6.848	0.159	16.911 ± 7.737	20.808 ± 5.531	0.061	18.684 ± 6.324	23.525 ± 3.648	0.003
1 M	16.268 ± 7.834	21.083 ± 5.120	0.020	17.090 ± 7.773	21.254 ± 4.970	0.039	18.384 ± 6.586	24.354 ± 3.174	< 0.001
3 M	15.247 ± 7.212	20.613 ± 6.064	0.011	16.237 ± 7.141	21.404 ± 4.852	0.007	17.658 ± 6.392	24.529 ± 3.420	< 0.001
6 M	15.684 ± 6.992	22.208 ± 5.203	0.001	16.426 ± 7.235	22.283 ± 5.154	0.004	17.590 ± 6.446	24.967 ± 3.081	< 0.001

*MRS* Mean Retinal Sentivity

### The changes of 4° MRS

Variations over time were not statistically significant as determined by ANOVA in the VA of 4° postoperative, but there was an interaction effect (F_time_ = 1.182, *p* = 0.334; F_group*time_ = 5.192, *p* = 0.001). In the placebo group, the MRS of 4° statistically significant reduction at each follow-up time (*p* = 0.001,0.002, < 0.001,0.006) and there was no statistically significant based on a pair-wise comparison(*p* > 0.05). However, it increased at each follow-up time point, and the differences were statistically significant (*p* = 0.016,0.006,0.004,0.001) in the combined group. Moreover, it increased significantly at 6 M compared with 1 M and 3 M in the combined group(*p* = 0.024,0.049). At 1 M, 3 M and 6 M, the MRS of 4° in the combined group was significantly better than that in the placebo group (t_1_ = -2.134, *p*_*1*_ = 0.039; t_3_ = -2.820, *p*_*3*_ = 0.007; t_6_ = -3.099, *p*_*6*_ = 0.004) (Table 3).

### The changes of 12° MRS

**Fig. 2:**
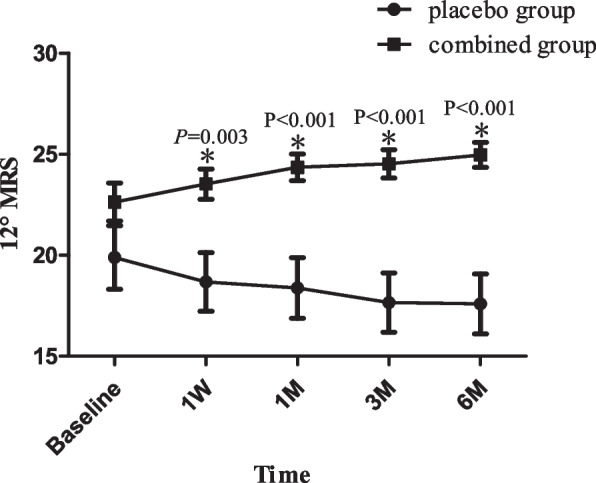
The MRS of 12° postoperative. The MRS of 12° of baseline and at 1 month,3 month and 6 months after surgery were shown. MRS: Mean Retinal Sentivity

**Fig. 3:**
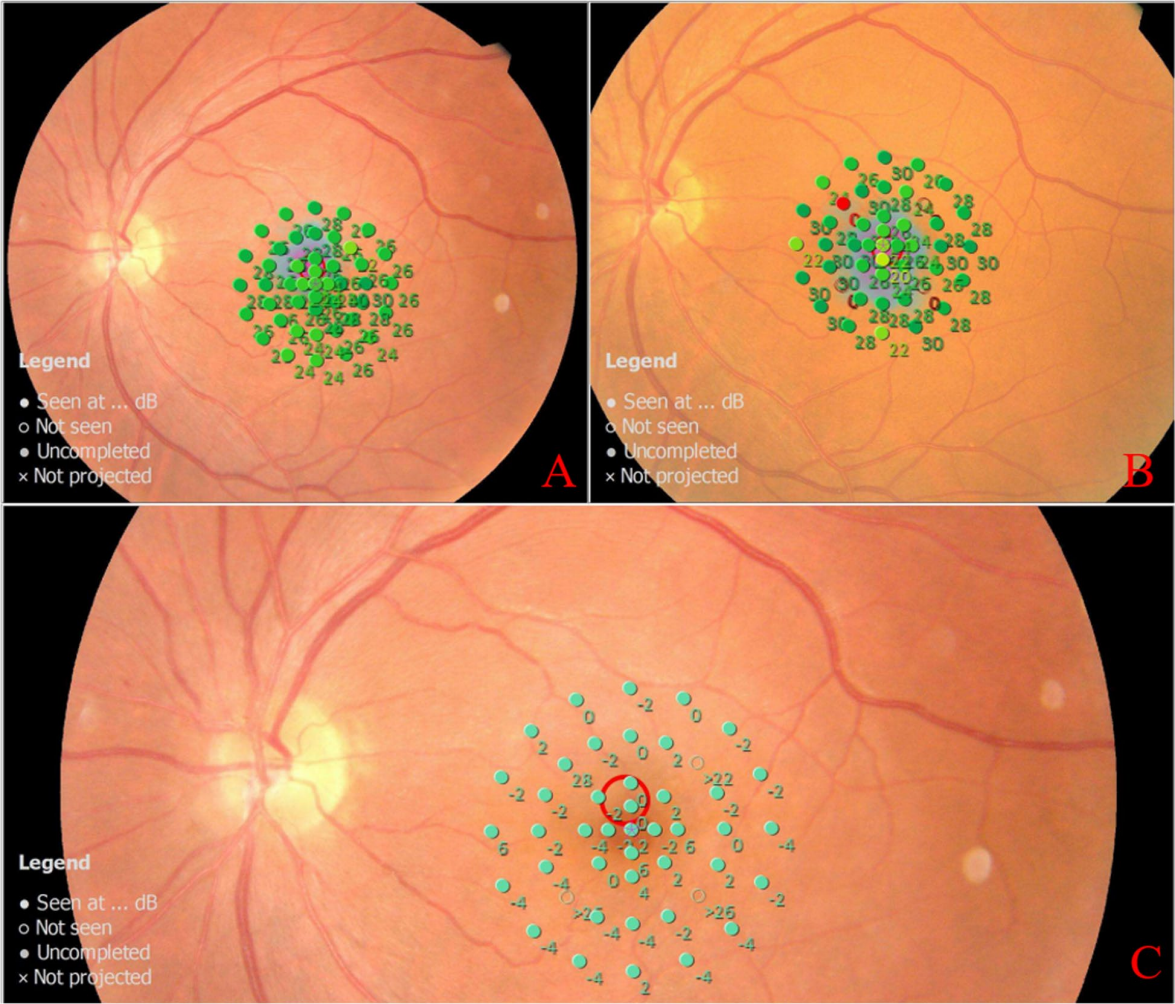
Representative microperimetry images for one eye in the placebo group before and after surgery. **A** and **D**: baseline; **B** and **E**: the 6-month post-surgery; **C** and **F**: the difference values between baseline and the 6-month post-surgery. The patient is a 60 years old man who had an eye with a idiopathic macular hole. Preoperatively, the mean retinal sensitivity in 2°, 4° and 12°was 22.7 dB,22.8 dB and 24.5 dB respectively. The mean retinal sensitivity in 2°, 4° and 12 was 21.3 dB,21.5 dB and 23.2 dB respectively in the 6-month postoperative

**Fig. 4:**
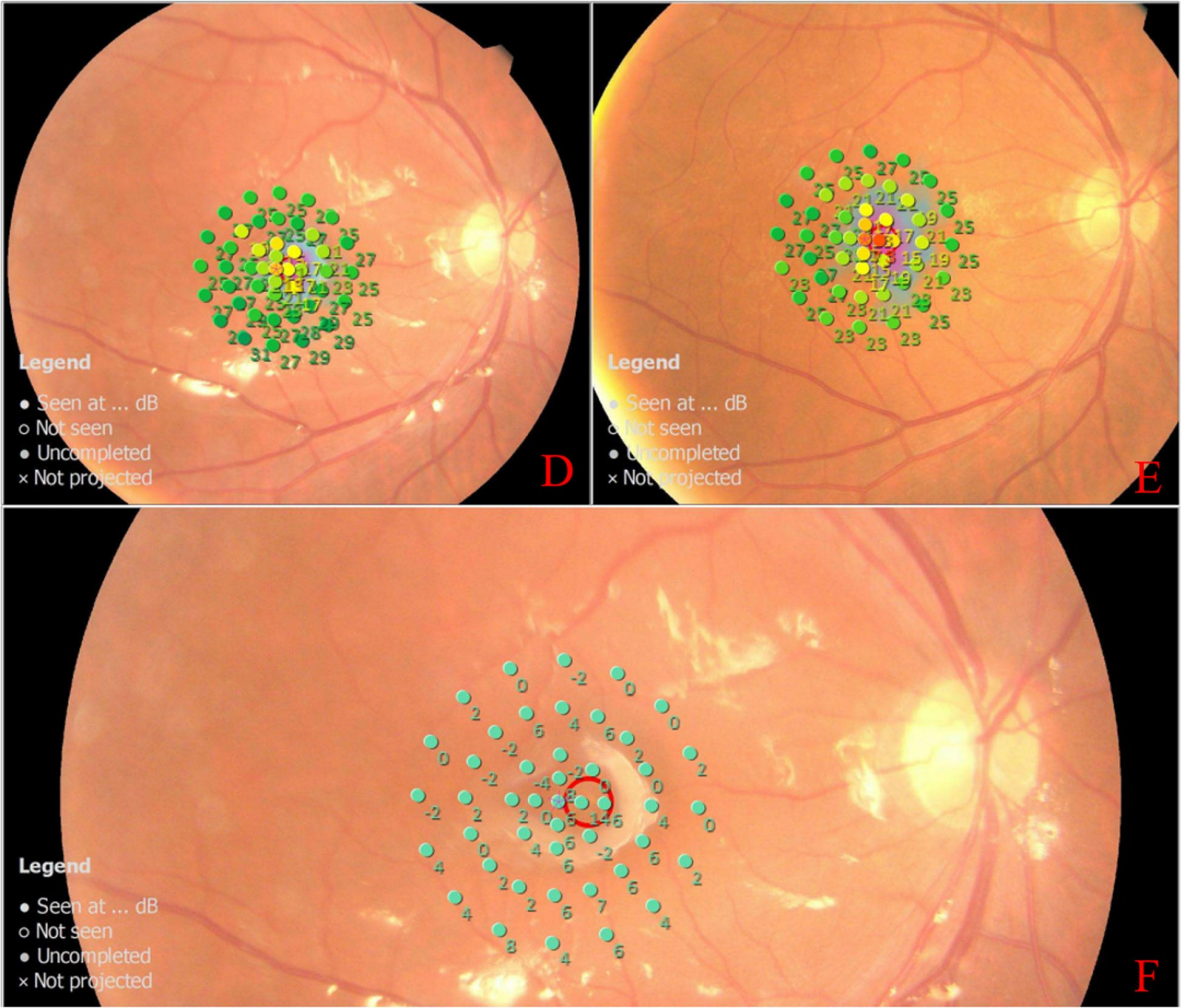
Representative microperimetry images for one eye in the combined group before and after surgery. The patient is a 56 years old woman who had an eye with a idiopathic macular hole. Preoperatively, the mean retinal sensitivity in 2°, 4° and 12°was 9.0 dB,12.7 dB and 19.6 dB respectively. The mean retinal sensitivity in 2°, 4° and 12 was 19.0 dB,18.1 dB and 24.2 dB respectively in the 6-month postoperative

The difference was statistically significant between groups and there was an interaction (F_group_ = 14.046, *p* = 0.001; F_group*time_ = 5.003, *p* = 0.002). The MRS of 12° decreased with time in the placebo group (F = 5.099, *p* = 0.009) and there was no statistically significant according to the pairwise comparisons(*p* > 0.05). The MRS of 12° improved significantly at 1 M, 3 M, and 6 M (*p* = 0.010, 0.019, 0.008) and according to the pairwise comparisons, the MRS of 12° at 1 M, 3 M and 6 M was significantly higher than that at 1W(*p* = 0.002,0.018,0.030) in the combined group. The MRS of 12° in the combined group was more obvious than that in the placebo group at each follow-up time point and the difference was statistically significant (t_1w_ = -3.151, *p*_*1*_ = 0.003; t_1M_ = -3.912, *p*_1_ < 0.001; t_3M_ = -4.521, *p*_3_ < 0.001; t_6M_ = -4.948, *p*_6M_ < 0.001) (Figs. 2, 3 and 4 and Table 3)).

### The change of EZ/ELM and correlation analysis

The integrity of ELM in combination group was better than that in placebo group at 6 M postoperative(*p* = 0.022) and that of ELM was preferable in the combined group at 3 M and 6 M after surgery(*p* = 0.012,0.004). Correlation analysis showed that the integrity of EZ at 1 M, 3 M and 6 M after surgery was correlated with 12°MRS (*r* = -0.318, -0.343,-0.322;*p* = 0.023,0.033, < 0.001**)**. There was no correlation between postoperative ELM integrity and postoperative BCVA and 12°MRS(*p* > 0.05).

## Discussion

The macular fovea has no inner retinal layers. It is formed by two populations Müller cells: specialized cells that formed Müller cell cones and Müller cells of the foveal walls and parafovea which had a characteristic z-shape [13]. The traction of the vitreous cortex cause Müller cell dysfunction in the fovea, which promotes the formation of cystic cavities in the fovea [14]. Nevertheless, the mechanical stress, generated by the hydrostatic pressure in the cavities, will lead to the enlargement of the cystic cavity. It can further aggravate the damage to Müller cells, promote the separation of Müller cells from the Outer Nuclear Layer (ONL), and cause the rupture of Müller cell cone, which lead to the formation of MH [15–17]. At present, IMH is mainly treated by surgery, and PPV is the basic surgical method for the treatment of IMH. With the development of technology, the application of PPV combined with ILM peeling, ILM tamponade, ILM transplantation, lens capsule transplantation, retinal transplantation and amniotic membrane transplantation technology have improved the anatomical and functional recovery of IMH to some extent [18–20]. Moreover, these technologies also had a certain effect on refractory and recurrent IMH. Nevertheless, each surgical method still has some limitations [21–23]. Some studies have reported that the tissue movement, mediated by Müller cells, generated fovea during ontogeny and also promotes MH closure and recovery of the structure of fovea [15]. In addition, Müller cells acts as stem cells in some mammals, which not only can rapidly differentiate into photoreceptor cells, but also have a protective effect on photoreceptors and Retinal Ganglion Cells (RGC) [24]. Under sufficient stimulation, new neurons are generated to restore vision through the NGF-Trka-PI3K-AKt-CREB signal transduction pathway [25]. Previous studies have indicated that the structure and function of the axon terminal of Müller cells were closely related to RGC. It can actively regulate Müller cells and promote the development of retinal nerve axons [26]. Therefore, it plays a protective role in idiopathic full-thickness MH. Recent studies have shown that PPV combined with ILM tamponade and intravitreal mNGF in the treatment of large MH can improve the microstructure of the macula and final BCVA [27]. In sum, adjuvant mNGF in the treatment of IMH has a good application prospect, but there is still a lack of abundant clinical evidence.

Our results show that compared with the preoperative, the perimeter and circularity of FAZ in two groups decreased with the change of the time. In the placebo group, no statistically significant changes in the vascular and perfusion density of SCP in the foveal region were found, and in the combined group, the vascular density of SCP increased at 1W and 6 M, the perfusion density of SCP was significantly improved at each follow-up time point that was consistent with the results of Tang et al. [28]. Studies have shown that compared with normal eyes, retinal sensitivity and microcirculation decreased in IMH eyes, and the reduction of visual acuity in the affected eyes is related to the changes of retinal microcirculation [29]. This contradicts with our results. As it is well known that the vascular and perfusion density of SCP mainly reflects the perfusion status of the micro-vessels in retina and the amount of blood perfusion within retinal vessels severally. This suggests that mNGF can improve the perfusion of retinal micro-vessels, increase the perfusion of retinal vessels, improve the ischemic state of the macular and the BCVA of patients with IMH.

Our results showed that compared to preoperative, the MRS of 2°, 4°, and 12° in the macula decreased in the placebo group. In contrast, the MRS of 2°,12°, and 4° in the macula increased at 1, 3, and 6 months, and each follow-up time point in the combined group severally. The difference was statistically significant. The MRS of 2°,4° and 12° in the combined group was better than that of the placebo group at 1 M, 3 M, 6 M and each follow-up time point respectively, and the difference was statistically significant. The effect on MRS of PPV combined with ILM peeling after IMH is still controversial. Kaluzny et al. [30] who used the microperimetry to measure the MRS of the ILM peeled area and the unpeeled area found that the MRS in the ILM peeled area was significantly reduced. It was hypothesized that it might be related to the damage of the retinal nerve fiber layer during ILM peeling. Tortuyaux et al. [31] revealed that there was no significant improvement of MRS after ILM peeling through a 10-year follow-up of IMH patients treated with PPV combined with ILM peeling. However, researches by Huang Ziqiong et al. proved that the MRS was improved compared with that before surgery [32]. What’s more, the reduction of retinal sensitivity and the thickness of ganglion cell complex did not cause a decline of MRS postoperative. Wang et al. [33] found that the MRS of the macula increased after ILM peeling, and it could be used as an index to predict postoperative BCVA. We hypothesized that the increase of MRS in the combined group was related to the protection of RGC by mNGF. Our conjecture is also consistent with the results of previous studies [34]. Our study showed that the ELM and EZ recovered after operation to some extent and the integrity of EZ at 1 M, 3 M and 6 M after surgery was correlated with 12°MRS. This is consistent with previous research [8, 35].

In conclusion, our study shows that PPV combined with ILM peeling and intravitreal injection of mNGF is more effective than PPV combined with ILM in the treatment of IMH by OCTA and MP-3 microperimetry, which can improve macular blood flow and patients' visual function. To the best of our knowledge, this is the first study using OCTA and microperimetry to investigate the changes of macular retinal blood flow and MRS after PPV combined with ILM peeling and intravitreal injection of mNGF for the treatment of IMH. In this study, we accurately and quantitatively analyzed the changes of macular blood flow and MRS after IMH.

This study still has some limitations: (1) our sample size is small and the follow-up time is short and the selection bias of retrospective study cannot be avoided. (2). The tamponade agent were air or silicon oil in the vitreous because of the lack of C3F8 which was better for the MH surgery at present. (3) The half-life of mNGF was short. It is not studied that injection mNGF into the vitreous to maintain the stable concentration of mNGF on IMH in this study. Long-term studies with large samples and the effects of mNGF on deep retinal and choroidal blood flow are the directions of our future research.

## Data Availability

The datasets used and/or analysed during the current study available from the corresponding author on reasonable request.
